# Antheridiogen controls spatial dynamics of sex expression in naturally occurring gametophytes of the tree fern *Cyathea multiflora*

**DOI:** 10.1002/ajb2.16040

**Published:** 2022-08-18

**Authors:** Aidan D. Harrington, Jennifer Blake‐Mahmud, James E. Watkins

**Affiliations:** ^1^ Plant and Microbial Biology Program, College of Biological Sciences University of Minnesota Twin Cities St. Paul MN 55108 USA; ^2^ Biology Department Hope College Holland MI 49423 USA; ^3^ Department of Biology Colgate University Hamilton NY 13346 USA

**Keywords:** antheridia, Cyatheales, hermaphrodite, ontogeny, outcrossing, pteridophyte

## Abstract

**Premise:**

Antheridiogen systems are a set of pheromonal mechanisms that control sex expression in fern gametophytes. However, antheridiogen has rarely been studied outside of the laboratory, and little is known about its function in natural settings. Combining predictions based on field and laboratory study, we tested whether the sexual structure of gametophytic colonies of a tree fern were attributable to antheridiogen.

**Methods:**

Gametophytic colonies of the antheridiogen‐producing tree fern *Cyathea multiflora* were collected at La Selva Biological Station in Costa Rica in January 2019. The sex of each gametophyte was determined, mapped, and spatial statistic approaches were used to examine the distribution of sex in each colony.

**Results:**

In all gametophytic colonies, males were most common, representing 62–68% of individuals. No hermaphroditic gametophytes were identified in any colony. A quadrat‐based method showed female gametophytes were not clustered in each colony, while male gametophytes were clustered. In two of the colonies, the *K*(*r*) test statistic for males was greater than expected compared to random simulations of sex expression, indicating male sex expression was spatially associated with females.

**Conclusions:**

This study provides the first documentation of spatial sex expression in natural settings of gametophytes of an antheridiogen‐producing tree fern species. The profound impact of antheridiogen on gametophytic sex expression in field settings suggests this system is intimately tied to mating system, fitness, and genetic diversity in *Cyathea multiflora*.

Studies of fern biology have long sought to reconcile the evolutionary success of the lineage with what was historically thought to be a limited reproductive system (Klekowski and Baker, 1966; Klekowski, 1969a, 1969b). This view was based on the unique reproductive biology of fern gametophytes compared to other plant lineages. In sporophyte‐dominant seed plants, gametophytes are strictly dioicous with sex determination always controlled by the sporophyte (Pannell, 2017). In gametophyte‐dominant bryophytes, approximately 60% of species have dioicous gametophytes with sex frequently determined by sex chromosomes (Villarreal and Renner, 2013; Glime and Bisang, 2017). In contrast, fern gametophytes live independently from fern sporophytes, lack sex chromosomes, and roughly 99% of species can produce monoicous gametophytes (Villarreal and Renner, 2013). The potential for self‐fertilization at the gametophytic stage in ferns (gametophytic selfing) presents a unique challenge for fern reproduction (Haufler, 2002; Haufler et al., 2016; Sessa et al., 2016), leading to the suggestion that ferns are habitual inbreeders but avoid the consequences of gametophytic selfing through polyploidy (Klekowski and Baker, 1966; Masuyama and Watano, 1990). While polyploidization does play a larger role in fern speciation compared to flowering plants (Wood et al., 2009), recent work refutes the idea that the widespread capacity for monoicy equals widespread inbreeding (Pelosi and Sessa, 2021).

In a meta‐analysis of the numerous population genetic studies done on fern sporophytes, Pelosi and Sessa (2021) report that most ferns are highly outcrossing. Experiments show that isolated gametophytes of many primarily outcrossing species can eventually become hermaphroditic and self‐fertilize, but the resultant homozygous sporophytes express all deleterious alleles and likely experience a high degree of inbreeding depression (Klekowski and Lloyd, 1968; Klekowski, 1969a; Lloyd, 1974; Haufler and Soltis, 1984; Peck et al., 1990; Soltis and Soltis, 1990; Haufler, 2002; Wang et al., 2012). Thus, gametophytic selfing only becomes prevalent under strong selective pressure, such as when long‐distance dispersal or high habitat patchiness limits mate‐finding (Baker, 1955; Lloyd, 1974; Soltis and Soltis, 1990; Barrington, 1993; de Groot et al., 2012a, 2012b; Hepenstrick et al., 2021). Outside of those conditions, ferns are primarily outcrossers, which is supported by several mechanisms including ontogenetic sequences and antheridiogen systems (Soltis and Soltis, 1990; Haufler, 2002; Haufler et al., 2016; Sessa et al., 2016).

Of the mechanisms thought to promote outcrossing in ferns, antheridiogen is one that has seen considerable interest. Antheridiogen broadly refers to a number of gibberellic acid derivatives that were first detected in laboratory cultures of gametophytes and are now known to be widespread across the phylogeny of ferns (Dopp, 1950; Näf et al., 1975; Yamane, 1998; Hornych et al., 2021). Antheridiogen, secreted by gametophytes once a notch meristem develops, has several functions including promotion of spore germination in the dark, induction of precocious antheridia development, suppression of gametophytic vegetative growth, and delay or prevention of gametophytes from becoming hermaphrodites (reviewed by Näf et al., 1975; Yamane, 1998). Concentrations as low as 10^−10^ M may induce antheridial formation (Tanaka et al., 2014) and can affect gametophytes at a distance of up to 7.5 cm from the source on soil (Greer and McCarthy, 1997). This system likely provides an evolutionary benefit by maintaining a dioicous gametophytic community that promotes outcrossing, though other frameworks including mate choice and sibling altruism merit investigation (Miller, 1968; Schedlbauer and Klekowski, 1972; Willson, 1981). While careful study has shown that antheridiogen strongly influences the sexual development of gametophytic communities in the laboratory, much less is known about the function of antheridiogen in natural gametophytic colonies, especially because the field environment imposes stochasticity not found in the laboratory (Schneller et al., 1990; Ranker and Houston, 2002).

Only three studies to date have explicitly studied the activity of antheridiogen in natural colonies of gametophytes (Tryon and Vitale, 1977; Schneller, 1979; Hamilton and Lloyd, 1991). These studies cover several antheridiogen‐producing species and suggest that gametophytic sex expression is modulated by antheridiogen in the field. Combined with the results of laboratory investigations, the results of these studies provide a framework to predict the function(s) of antheridiogen in natural gametophytic colonies. The first prediction is that gametophytic colonies with active antheridiogen will have many small antheridia‐bearing gametophytes, some larger archegoniate gametophytes, and few hermaphroditic gametophytes possessing both antheridia and archegonia. This prediction is based on the sexual ontogeny of fern gametophytes (Atkinson and Stokey, 1964; Klekowski, 1969a; Soltis and Soltis, 1990; Greer et al., 2009). Isolated or early germinating spores of antheridiogen‐producing taxa in the Schizaeales and core leptosporangiate lineages develop into female gametophytes first before eventually becoming hermaphrodites (Klekowski, 1969a, 1969b; Banks, 1999; Greer et al., 2009). These older gametophytes exude antheridiogen that promotes the development of males among ungerminated or later arriving spores in the colony (Schneller, 1979, 1988; Schneller et al., 1990; Atallah and Banks, 2015). If obtained, hermaphroditism develops in the oldest gametophytes that were previously unisexual female gametophytes and, in some examples, on gametophytes that reproduced vegetatively from older gametophytes (Stokey, 1930; Atkinson and Stokey, 1964; Banks, 1999; Atallah and Banks, 2015). Alternatively, lineages of ferns outside the core leptosporangiate clades and Schizaeles typically develop as male first before maturing into hermaphrodites or initiate gametangia simultaneously, so antheridiogen in these groups functions differently (Greer et al., 2009). In a study of *Osmundastrum cinnamomeum* (Osmundaceae) gametophytes, which possess a male‐first ontogeny, it was found that gametophytes may skip male‐first expression for female‐first expression in culture. The female gametophytes then suppressed the further maturation of the remaining male gametophytes in the colony, demonstrating a putative antheridiogen system (Hollingsworth et al., 2012). While multiple mechanisms may drive the increase of female gametophytes in multi‐spore cultures of *O. cinnamomeum* (Hollingsworth et al., 2012), the suppression of male maturation via a putative antheridiogen system clearly results in more unisexual gametophytes and fewer hermaphrodites. Whether a species possesses a regular or *Osmundastrum*‐type putative antheridiogen system, the expectation is that colonies of gametophytes where sex is affected by antheridiogen will have few hermpahrodites and an abundance of unisexual gametophytes. This prediction is consistent with observations from the limited number of field studies of antheridiogen‐producing species, where few hermaphrodites were found (Tryon and Vitale, 1977; Schneller, 1979; Hamilton and Lloyd, 1991).

In principle, the presence of small unisexual male and large unisexual female gametophytes would be a strong indicator of antheridiogen activity. This pattern is seen repeatedly in numerous lab studies where mature gametophytes are grown with younger gametophyte or placed in spore cultures (Voeller, 1964a; Hickok, 1983; Peck et al., 1990; Takeno, 1991; Warne and Hickok, 1991; Fernandez et al., 1997; Chiou et al., 2002; Greer and Curry, 2004; Prada et al., 2008; Hornych et al., 2021). However, environmental factors also impact sexual development (Rubin and Paolillo, 1983; Pangua et al., 1994; Ranker and Houston, 2002; Quintanilla et al., 2007; DeSoto et al., 2008; Atallah and Banks, 2015; Pajarón et al., 2015). For example, some studies show that environmental factors that slow growth may induce male‐first expression in the absence of antheridiogen systems even in species with a female‐first ontogeny (Haufler and Soltis, 1984; Hamilton and Lloyd, 1991; DeSoto et al., 2008). Thus, the presence of size‐based sexual structure in a natural colony may not always indicate the functioning of an antheridiogen system. We still lack a clear understanding of all the mechanisms that promote unisexual males that is comprehensive across lineages with differing ontogenetic sequences (Klekowski, 1969a; DeSoto et al., 2008; Greer et al., 2009; Hollingsworth et al., 2012; Pajarón et al., 2015). Below, we develop a second prediction that further differentiates colonies of gametophytes with antheridiogen from those without.

The second prediction is that antheridiogen activity results in nonrandom spatial arrangement of sex expression at the scale of a single gametophytic colony (Schneller et al., 1990; Hamilton and Lloyd, 1991). Antheridiogen must be secreted by some gametophytes and detected by others, making the distance between gametophytes an important factor for the success of an antheridiogen system (Tryon and Vitale, 1977; Greer and McCarthy, 1997; Greer et al., 2009; Tanaka et al., 2014). The antheridiogen concentration decreases as a function of distance in the substrate, suggesting that the likelihood of male sex expression similarly decreases with distance from the antheridiogen source (Tryon and Vitale, 1977; Greer and McCarthy, 1997). Consequently, antheridiogen activity in a colony of gametophytes is predicted to produce a pattern where male sex expression is dependent on proximity to female gametophytes. This prediction is important because if sex expression is not modulated by antheridiogen (e.g., controlled by microclimate or competition), then male sex expression would not be strictly associated with the presence of females.

Here we examined natural colonies of gametophytes of the tree fern *Cyathea multiflora* (Cyatheaceae) with respect to our two predictions. Only one field study has demonstrated antheridiogen activity in tropical species (*Asplenium pimpinellifolium* and *Lygodium heterodoxum*), and none have investigated gametophytes of tree ferns (Tryon and Vitale, 1977). Moreover, antheridiogen systems have only recently been discovered in tree ferns, including *C. multiflora* (Hornych et al., 2021). However, the type of antheridiogen system operating in *C. multiflora* is unclear. While previous studies repeatedly showed that gametophytes of the Cyatheales broadly possess the ancestral male to hermaphroditic ontogeny and reported no evidence for antheridiogen systems in any Cyatheales species (Näf and Trager, 1960; Voeller, 1964b; Weinberg and Voeller, 1969;  Nayar and Kaur, 1971; Huang et al., 2001; Chiou et al., 2003; Khare et al., 2005, 2009; Rechenmacher et al., 2010), Hornych et al. (2021) found that *C. multiflora* proceeded through a female to hermaphrodite ontogeny when grown in culture. Antheridia formation was initiated before archegonia only if a mature, female, conspecific gametophyte was present, indicating an antheridiogen system (Hornych et al., 2021). Given the previously reported male‐first ontogeny of *Cyathea* gametophytes, the results of Honrych et al. (2021) might be explained by the functioning of an *Osmundastrum*‐type putative antheridiogen system, where gametophytes that are typically male‐first develop as female‐first in favorable conditions and then suppress further maturation of other gametophytes with antheridiogen (Greer et al., 2009; Hollingsworth et al., 2012). Another report of female‐first ontogeny in the Cyatheaceae comes from Stokey (1930), who observed that some vigorous gametophytes develop archegonia only, supporting the idea that culture conditions may induce female‐first development in species with an otherwise male‐first ontogeny. Alternatively, *Cyathea multiflora* may simply represent an independent origin of the female‐first ontogenetic sequence more commonly observed among core leptosporangiate clades. In either case, the results of Hornych et al. (2021) provide compelling evidence that at least some species of *Cyathea* possess some kind of antheridiogen system that might be detectable in a field setting.

We hypothesized that if natural groups of *Cyathea multiflora* gametophytes include many hermaphroditic gametophytes and if sex expression is randomly spatially distributed in the clusters, then antheridiogen is likely not effective or active. Conversely, colonies structured by antheridiogen will have male‐biased sex ratios and few to no hermaphrodites. Male sex expression will be spatially associated with female gametophytes, which will be randomly dispersed. The extent to which field colonies of antheridiogen‐producing gametophytes conform to both these predictions is not entirely certain, but the few studies in natural settings to date suggest that these are reasonable criteria for evaluating the presence of antheridiogen activity in nature.

## MATERIALS AND METHODS

### Study site and gametophytic colony identification

We conducted the study in January 2019 at La Selva Biological Station in Sarapiqui, Costa Rica, a lowland tropical rainforest. We located gametophytes of *Cyathea multiflora* on a large tip‐up mound, elevated by the roots of a fallen tree at the beginning of the Arboretum trail (10°25′49.3″N, 84°00′23.0″W). To delimit individual colonies, we selected gametophytes growing in distinct, and as much as possible, isolated groups that could be entirely encapsulated by a 10‐cm‐diameter petri dish. We ignored colonies on non‐uniform substrates or that crossed a clear division in light or water to limit confounding microclimate‐induced differences in sex expression. After photographing each colony, we collected the entire colony with the substrate intact by inserting the petri dish into the soil around them and removing the cluster, keeping all gametophytes in the same orientation. To determine sex expression and confirm species identification, we removed gametophytes from the substrate, then wet‐mounted and examined them with an Olympus BX41TF optical microscope (Evident Scientific, Tokyo, Japan) and bright field optics at 40× magnification for the presence of archegonia, antheridia, and characteristic multiseriate hairs found only on *Cyathea* gametophytes. We identified gametophytes by following a developmental series from young gametophytes to mature gametophytes with sporophytes attached, a method commonly used to identify fern gametophytes (Lagomarsino et al., 2012; Canestraro et al., 2014; Watkins and Moran, 2019; Watts et al., 2019; Harrington and Watts, 2021). *Cyathea* gametophytes and young sporophytes both have diagnostic multicellular hairs (Atkinson and Stokey, 1964; Momose, 1968; Nayar and Kaur, 1971; Chen et al., 2008). Sporophytes were identified to species using the key in *Flora Mesomamericana* (Davidse et al., 1995). Together, these characters supported identification of the gametophytes, young sporophytes attached to gametophytes, and mature sporophytes in the surrounding area all as *C. multiflora*. We excluded and ignored gametophytes that were glabrous and typical. Using the photographs, we mapped points onto the gametophytes digitally with ImageJ to obtain relative coordinate positions (Schneider et al., 2012). Unmarked gametophytes in the images were not identifiable as *Cyathea multiflora*. We then joined the mapped coordinates with the corresponding sex for each gametophyte.

### Statistical analysis

Gametophytic sex distributions within colonies were compared with a *G*‐test of goodness‐of‐fit. Sex distribution across the colonies was compared with a *G*‐test of independence. To test for spatial relationships between gametophytes, we first developed an informal model in terms of random and nonrandom spatial mechanisms. For all possible locations of a gametophyte in a hypothetical colony (e.g., wherever spores landed), female gametophytes were assigned to a subset of those locations by a random process (the spores which arrived or germinated first), and the sex of the remaining gametophytes is assigned to the remaining possible locations conditional on a nonrandom spatial process (detection or nondetection of antheridiogen). This general model predicts that the distribution of females within the colony is spatially random and that the distribution of males is spatially nonrandom *and* conditionally dependent on the presence of females.

First, we tested whether the gametophytes fit the general patterns hypothesized under this model using a quadrat counting method. The test takes the null hypothesis that the empirical distribution of gametophyte sex expression is a realization of a uniform Poisson process, a standard representation for a random distribution of points (Baddeley et al., 2015). Each combination of colony and sex was considered on its own and subdivided into the maximum number of quadrats possible while still containing at least five individuals in each. Gametophyte counts per quadrat were compared to expectations under the uniform Poisson process with a *χ* ^2^‐test. A significant result occurs when more gametophytes of a given sex than expected are observed within the quadrats, indicating clustering. This test was only performed with the alternative hypothesis of clustering, so some gametophytes not displaying clustering may have uniform distributions. While this test allowed us to confirm or reject clustering on a sex‐by‐sex basis, an additional test is needed to determine whether male sex expression is related to the presence of female gametophytes.

To test whether male sex expression is related to female proximity, we used a random labelling method and Ripley's *K*‐function *K*(*r*) adapted from Baddeley et al. (2015). An empirical test statistic is calculated as the difference in *K*(*r*) of the subset of gametophytes (male or asexual) from the *K*(*r*) of the subset and females together at increasing radii. Values greater or lesser than zero indicate that the clustering or dispersion, respectively, of the subset of gametophytes is associated with the presence of the female gametophytes. Significance of the test statistic is determined by comparing it to a null test statistic based on *K*(*r*) values calculated in the same way following random simulations of sex in each colony. Sex expression is simulated randomly 39 times such that the empirical data represents 1 of 40 possible realizations of sex expression in the colony (e.g., 5% of realizations). Thus, when the empirical *K*(*r*) values are greater than the envelope of simulations, then clustering in the tested subset is significantly associated with the presence of the female gametophytes at the *P* < 0.05 level. Conversely, if the empirical test statistic is below the envelope of simulations, then the presence of female gametophytes is associated with greater dispersion than random. Data were analyzed using R v4.0.5 “Shake and Throw” and the R package “spatstat” (Baddeley et al., 2015; R Core Team, 2021).

## RESULTS

### Identification of *Cyathea multiflora* gametophyte and autecological observations

**Figure 1:**
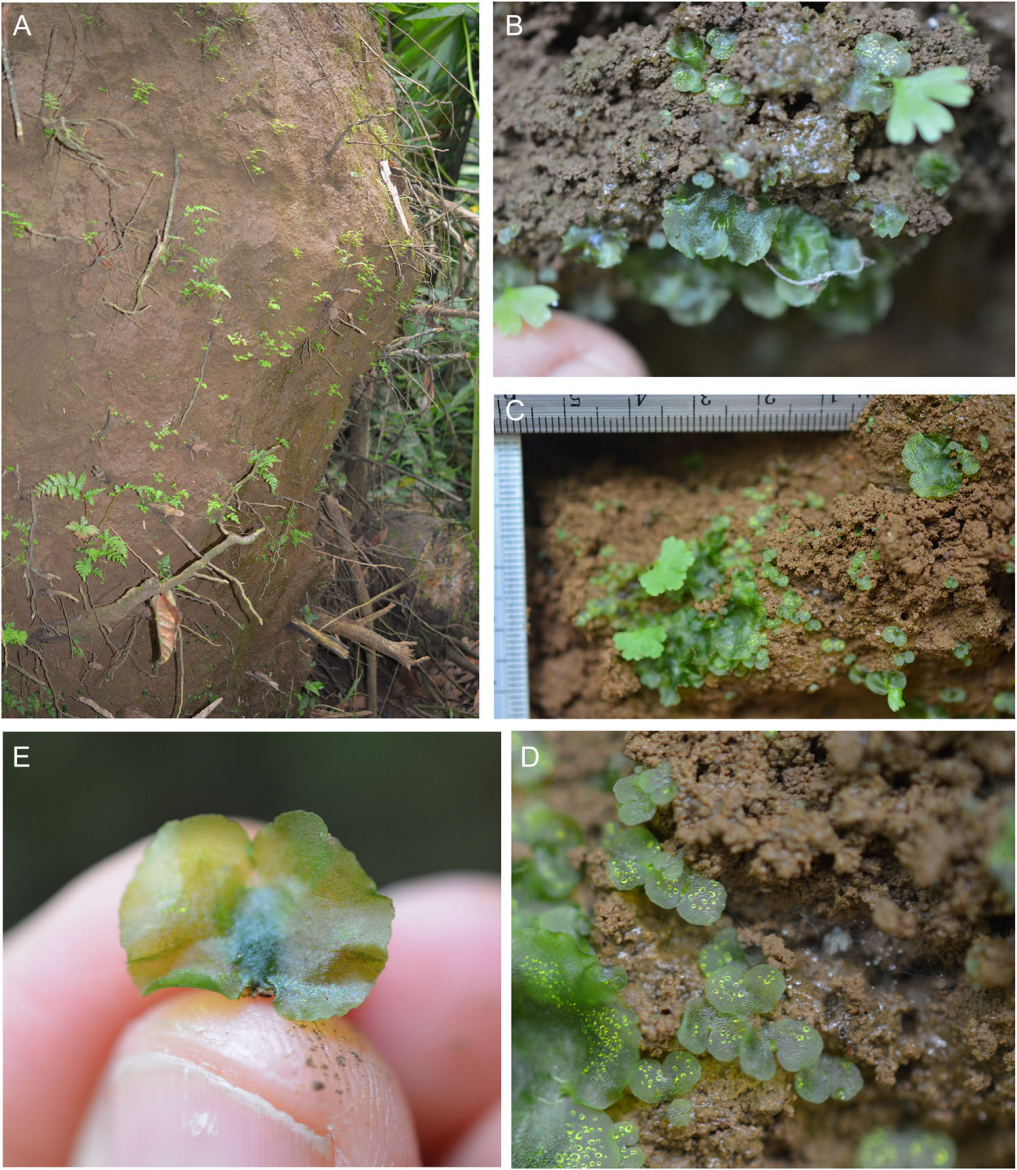
Habitat and gametophytic colonies. Clockwise from top left: (A) Tip‐up mound at La Selva Biological Station in Costa Rica where *Cyathea multiflora* gametophytes were located. (B–D) Detail images showing typical variation in size, shape, and reproductive state of gametophytes in the same community. (E) Individual mature female gametophyte.

**Figure 2:**
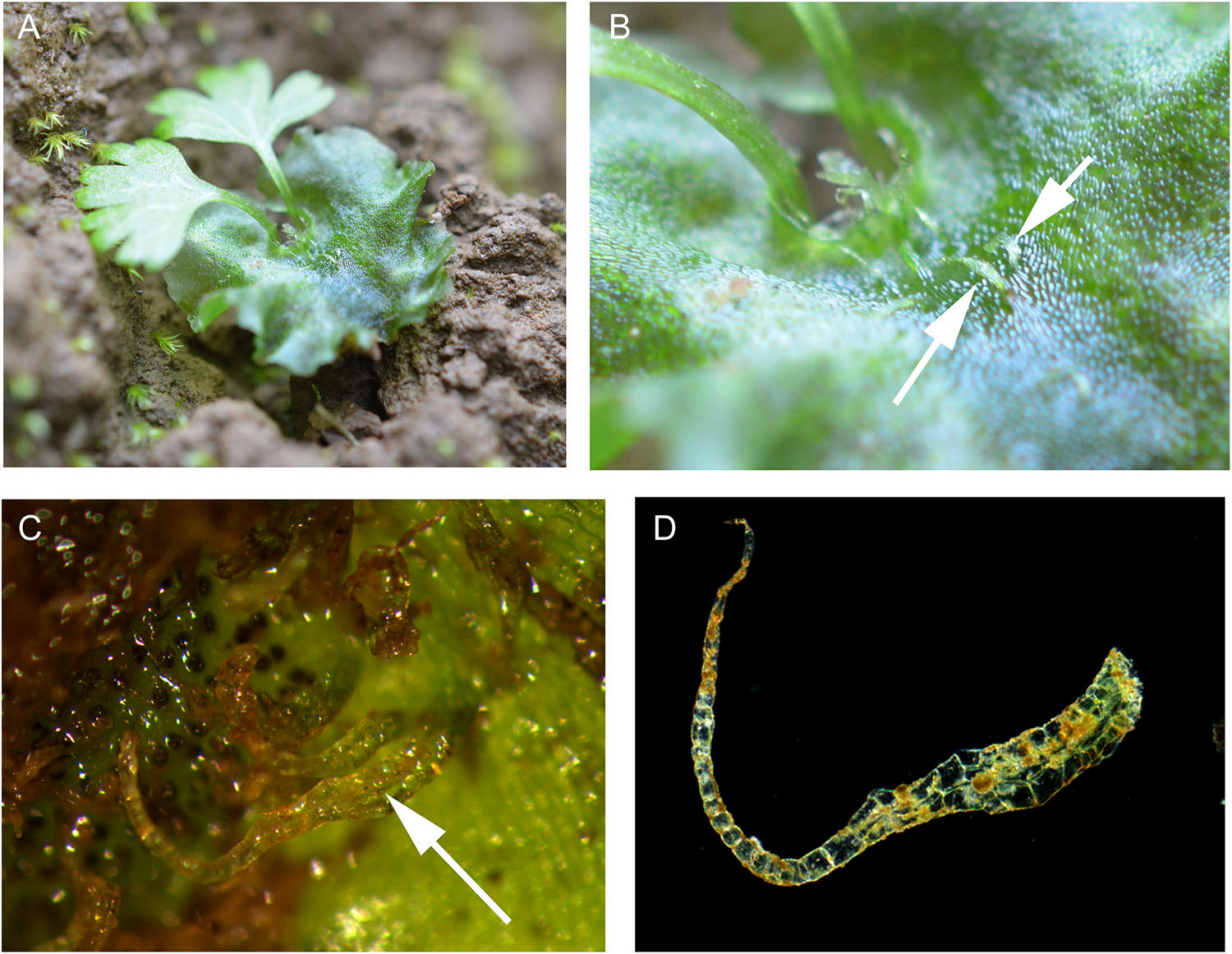
Identification of gametophytes. Clockwise from top left: (A) Young *Cyathea multiflora* sporophyte emerging from a fertilized gametophyte. (B) Characteristic multiseriate hairs on the gametophytes of *C. multiflora* as indicated by arrows. (C) Hair excised from the gametophyte. (D) Detailed view of a multiseriate hair (arrow) amidst archegonia.

**Figure 3:**
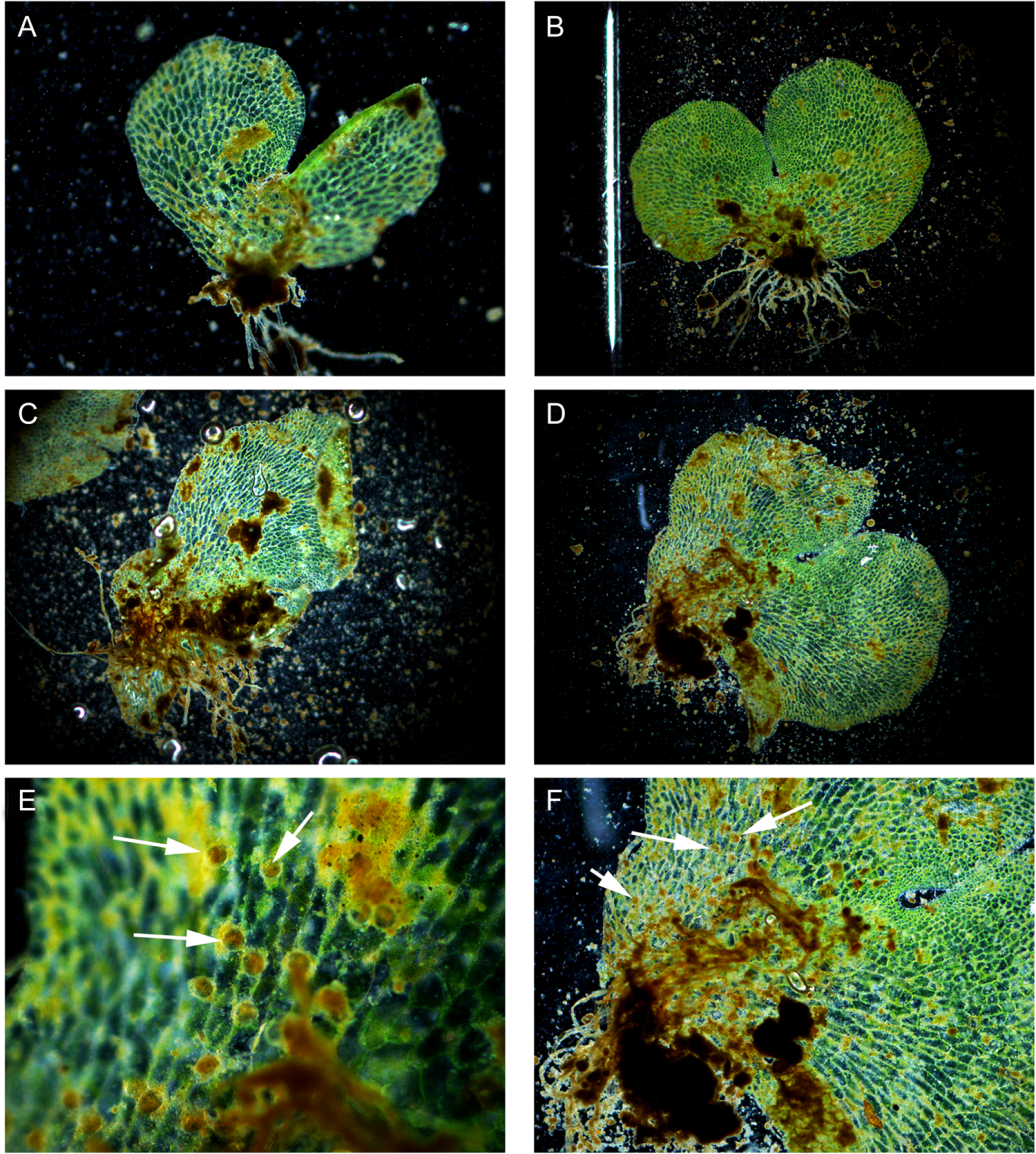
Determination of male gametophytes. (A–D) Male gametophytes of *Cyathea multiflora*. (E, F) Antheridia (arrows) on male gametophytes used to determine sex.

We discovered many thousands of gametophytes on a massive tip‐up mound from a recently fallen tree (Figure 1A). We were able to confidently identify several isolated clumps of *Cyathea multiflora* gametophytes (Figure 1B–E) by assessing the presence of the characteristic multiseriate and multicellular hairs that are found only in *Cyathea* (Figure 2A–D) and following the developmental series of gametophytes to *C. multiflora* sporophytes (Atkinson and Stokey, 1964). We found colonies of mixed sexes and easily identified both cordiform and spathulate male *C. multiflora* gametophytes in our study colonies (Figure 3). Many of the co‐occurring taxa on this and other tip‐up mounds at this site were early successional taxa that also have diagnostic hairs such as capitate glandular hairs in *Pityrogramma* and members of the *Dennstaedtiaceae* and acicular hairs of thelypteroid taxa. These characters allowed for easy elimination of other common species at the site.

### Gametophytic colonies and sex ratios

**Figure 4:**
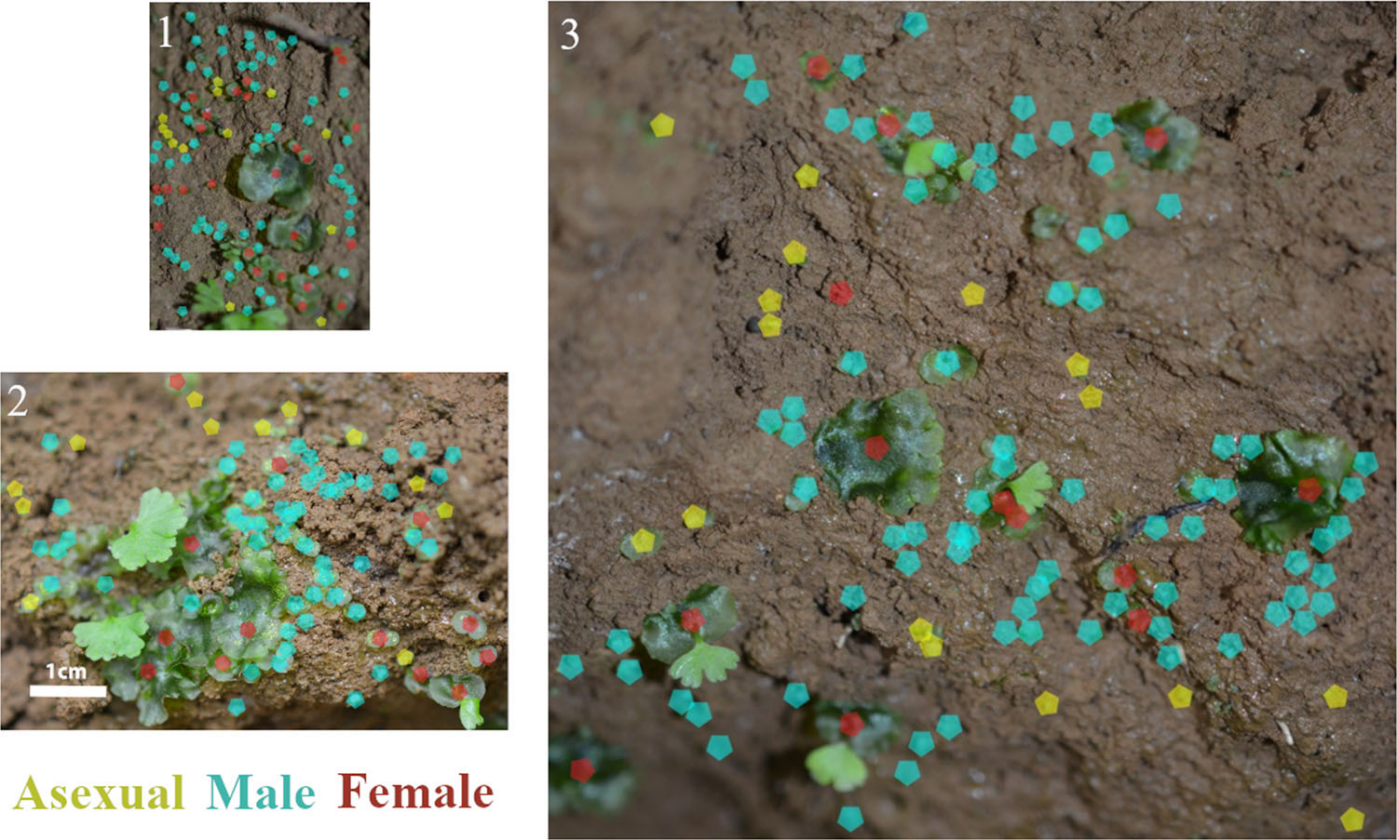
Study Colonies 1–3 of *Cyathea multiflora* gametophytes. Colonies are mapped with points representing the sex of the gametophyte. Red dots: female (archegoniate) gametophytes; blue dots: male (antheridial) gametophytes; yellow dots: asexual (no sex organs). All colonies are shown to scale; scale bar applies to all images.

We surveyed three separate gametophytic colonies of *Cyathea multiflora*, containing a total of 322 gametophytes (Figure 4A–C). Colonies were found on the bare soil of a large tip‐up mound and ranged in size from 12 to 81 cm^2^. Across all colonies, male gametophytes (63% of the total) were significantly more common than female (20%) and asexual (17%) gametophytes (*G* = 119.34, df = 2, *P* < 0.001) (Table 1). The distribution of sexes was not significantly different across colonies (*G* = 4.21, df = 4, *P* = 0.377). We did not identify hermaphroditic gametophytes in any colony. While size was not measured, female gametophytes were qualitatively much larger than male or asexual gametophytes, which varied in size. The largest gametophytes in each colony were exclusively female. Occasional female gametophytes were also attached to sporelings, indicating successful intergametophytic mating.

**Table 1:** Gametophytic colonies and sex ratios.

Colony	Female	Male	Asexual	Total
1	25 (0.26)	60 (0.61)	13 (0.13)	98
2	13 (0.17)	51 (0.68)	11 (0.15)	75
3	27 (0.18)	92 (0.62)	30 (0.20)	149
Total	65 (0.20)	203 (0.63)	54 (0.17)	322

*Notes*: Quantities of *Cyathea multiflora* gametophytes of each sex from each colony. Numbers in parentheses refer to the fraction of the total in that colony represented by each sex.

### Clustering of gametophytes

The quadrat test with a *χ* ^2^ counting method did not indicate clustering for female or asexual gametophytes in any colonies (Female 1: *χ* ^2^ = 0.44, df = 3, *P* = 0.932; Asexual 1: *χ* ^2^ = 3.77, df = 3, *χ* ^2^ = 0.052; Female 2: *χ* ^2^ = 0.08, df = 1, *P* = 0.782; Asexual 2: *χ* ^2^ = 0.09, df = 1, *P* = 0.763; Female 3: *χ* ^2^ = 3.67, df = 3, *P* = 0.229; Asexual 3: *χ* ^2^ = 6.00, df = 3, *P* = 0.116) (Table 2). Males were detected as clustering in Colony 2 and 3 (Male 2: *χ* ^2^ = 24.71, df = 8, *P* = 0.002; Male 3: *χ* ^2^ = 51.30, df = 15, *P* < 0.001) but not Colony 1 (Male 1: *χ* ^2^ = 12.40, df = 11, *P* = 0.334) (Table 2).

**Table 2:** Quadrat test of complete spatial randomness.

Colony	Sex	*χ* ^2^	df	*P*
1	M	12.40	11	0.334
	F	0.44	3	0.932
	A	3.77	1	0.052
2	M	24.71	8	0.002*
	F	0.08	1	0.782
	A	0.09	1	0.763
3	M	51.30	15	<0.001*
	F	3.67	3	0.229
	A	6.00	3	0.116

*Notes*: Summary of quadrat test for complete spatial randomness (M = male, F = female, A = asexual). Asterisk (*) denotes significance at the 0.05 confidence level. A significant result indicates clustering of gametophytes.

### Spatial associations with female sex expression

**Figure 5:**
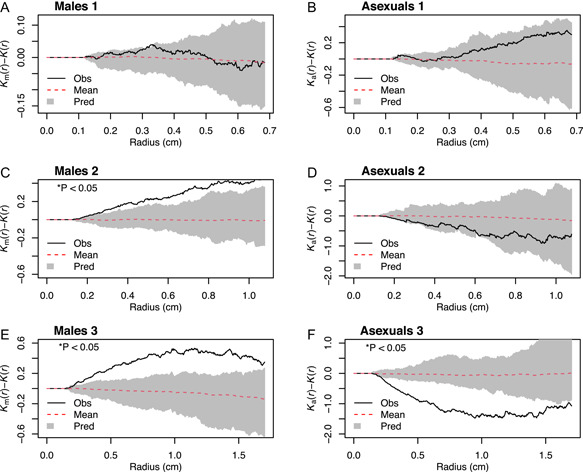
Results of *K*(*r*) algorithm and simulations. The *y*‐axis of each plot is the *K*(*r*) test statistic, and the *x*‐axis is the distance from female gametophytes in centimeters. Gray color (pred = prediction) represents the envelope based on random simulations of sex in each colony (red‐dashed line: mean of simulations), within which the empirical test statistic (black line, obs = observed) is considered not significantly different from random. (A, B) Colony 1 male and asexual empirical test statistics do not exceed the envelope. (C, D) Colony 2 males are above the envelope, indicating female gametophytes cause males to occur greater than chance. Asexuals display no significant pattern. (E, F) Colony 3 males are above the envelope and asexuals are below the envelope, indicating that female gametophytes are associated with the clustering of male gametophytes and dispersion of asexual gametophytes.

Results for the *K*(*r*) test statistic with randomized simulations of sex expression are shown in Figure 5A–F. The empirical test statistic did not exceed the 95% simulation envelope for males in Colony 1 (Figure 5A). Thus, Colony 1 did not exhibit significant spatial dependence on female gametophytes for male sex expression. The empirical test statistic did exceed the 95% simulation envelope for males in Colony 2 and 3 (Figure 5C, E), indicating clustering and spatial association with female gametophytes. These results are consistent with the patterns identified in the quadrat test. Asexuals in Colony 3 had significant spatial association with female gametophytes in the negative direction, suggesting that asexual gametophytes tend to be more dispersed away from female gametophytes than random in this group (Figure 5F).

## DISCUSSION

In this study, we provided evidence of an antheridiogen system in natural colonies of *Cyathea multiflora* gametophytes. Our results are consistent with the predictions that colonies structured by antheridiogen will have male‐biased sex ratios with few to no hermaphroditic gametophytes and spatial association of male sex expression with females. Our results further support previous studies that suggest that antheridiogen functions in natural gametophytic colonies and provide the first field evidence for a putative antheridiogen system in *Cyathea*.

### Prediction 1: Sex distribution and gametophytic colonies

Our results are consistent with the existing field studies where antheridiogen activity was confirmed. Hamilton and Lloyd (1991) found only 1.6% of *Polystichum acrostichoides* gametophytes were hermaphroditic across 28 gametophytic communities. While their study found different proportions of each sex versus the present study (asexuals 53%, males 39%, and females 6%), they included two‐celled gametophytes, which explains the increased representation of asexual gametophytes (Hamilton and Lloyd, 1991). Schneller (1979) found approximately 8% of *Athyrium felix‐femina* gametophytes were hermaphrodites in the field, males represented 50%, and the remaining gametophytes were evenly split between asexual and female. In several additional field studies of known antheridiogen‐producing taxa, only sex distributions were measured. In two *Sadleria* species, 0–12% of gametophytes were hermaphroditic with males representing the majority (Ranker and Houston, 2002). In *Blechnum spicant*, hermaphrodites comprised up to 8% of natural colonies early in the growing season but increased in frequency throughout the year because both sexes can mature into hermaphroditic gametophytes (Cousens, 1979; Cousens, 1981). In semi‐natural studies in which antheridiogen‐producing gametophytes were grown in the laboratory on soil substrates, hermaphrodites were also found to be highly uncommon (Schedlbauer and Klekowski, 1972; Schneller, 1988; Greer, 1993; Greer and McCarthy, 1997). In contrast, frequencies of hermaphrodites as high as 95% have been reported in natural colonies of species that do not produce antheridiogen (Peck et al., 1990). Collectively, our results and others confirm that a relative lack of hermaphroditic gametophytes and male‐biased sex ratios is highly characteristic of gametophytic colonies in antheridiogen‐producing species.

Despite differences in colony size and density, there was also consistency in sexual structure across our study groups. Colony 3 covered a much larger area than Colony 1 (81 cm^2^ vs. 12 cm^2^) but did not contain proportionally more gametophytes (*n*_3_ = 149 vs. *n*_1_ = 95), resulting in a 5‐fold difference in density between the two colonies. Despite this, the distribution of sexes in each colony was not significantly different. Because the natural soil environment is exposed to the elements and physically uneven, one might not predict equal conveyance and detection of antheridiogen throughout the substrate, especially if the gametophytes are spread out (Greer and McCarthy, 1997; Ranker and Houston, 2002). In our study, the presence of asexual gametophytes could reflect uneven antheridiogen distribution. Nonetheless, if antheridiogen induces a response at exceedingly low concentration and is readily spread through the soil, a colony of gametophytes would need to be significantly less dense than documented here before antheridiogen would become ineffective at modulating sex expression (Greer and McCarthy, 1997; Tanaka et al., 2014).

### Prediction 2: Clustering and spatial association of male sex expression

Our results mostly confirm the model we developed under prediction two, that males are clustered and associated with the presence of females. Tests for clustering revealed that males in Colonies 2 and 3 were clustered, males in Colony 1 were not clustered, and nonmales in all colonies were also not clustered (Table 2). The tests also showed that female gametophytes were randomly distributed within each colony, consistent with the randomness of the distribution of the spores on the soil (Table 2). Because the soil environment is devoid of antheridiogen at this stage, the first spores to germinate likely became female gametophytes, either because this is the typical sexual phenology of *C. multiflora*, or because minimal competition and vigorous growth favor archegonia to mature first (Stokey, 1930; Schedlbauer and Klekowski, 1972; Schneller, 1988; Schneller et al., 1990).

The results show that male clustering in Colonies 2 and 3 is spatially associated with females, indicating antheridiogen function (Figure 5C, E). Colony 1, however, lacks this signal, and there are several explanations (Figure 5A). Stokey (1930) reports that in low light, gametophytes of *Cyathea arborea* became elongated and male, eventually changing to female and back to male again. Such labile sex expression, especially in response to light and other environmental factors, is not well understood. Another possibility is that because each gametophyte was marked at its center and these points were used as the basis for analysis, large female gametophytes inflate the distances between females and the nearest observed males. Finally, there is evidence for variation in antheridiogen sensitivity both at the individual and population levels, which could explain some variation across colonies that contain a mixture of genotypes (Schneller et al., 1990). Nonetheless, male sex expression is primarily a function of proximity to females in our two largest colonies by area, agreeing with the results of other field studies that examined distances between gametophytes (Tryon and Vitale, 1977; Hamilton and Lloyd, 1991).

Spatial patterns of asexual gametophytes are also important to consider. None displayed significant clustering in the quadrat test, though clustering of Colony 1 was nearly significant (Table 2). Proximity to females either had no or significantly negative effects on the probability of asexual sex expression (Figure 5F). In Colonies 2 and 3, asexuals are dispersed toward the periphery, aligning with the expectation that they represent a failure to detect antheridiogen as they tend to be further from the female gametophytes. In Colony 1, most asexual gametophytes occurred in a single, localized group, perhaps reflecting uneven antheridiogen distribution in the soil. Given that the sexual ontogeny for *C. multiflora* is female‐first in culture conditions, it is also possible that the presence of maturing, cordate, asexual gametophytes are among the more vigorous individuals which will bypass antheridia production for archegonia production. Alternatively, asexual gametophytes could be the product of high competition, as found by Huang et al. (2004), or simply are individuals that have not yet produced antheridia.

### Evidence for antheridiogen in *Cyathea multiflora*

Our results in combination with Hornych et al. (2021) suggest that *C. multiflora* possesses an antheridiogen system, similar to the putative system in *Osmundastrum cinnamomeum* found by Hollingsworth et al. (2012). In species with this system, sex among initially germinating gametophytes is determined primarily by growth conditions, or possibly a genetic polymorphism (Hollingsworth et al., 2012). The uniform development of *C. multiflora* gametophytes as female‐first in Hornych's study (which used multiple maternal spore sources) suggests that a genetic polymorphism is not responsible for determination of female sex. Assuming *C. multiflora* does not represent an independent origin of a female‐first ontogeny in the Cyatheales, females are likely the product of an environmental sex determination mechanism where vigorous growth due to a lack of competition and/or an abundance of resources (e.g., a low‐density culture) prompts the development of archegonia first. Several studies have shown competition is a crucial factor for sex expression irrespective of an antheridiogen system because size is closely related to the ability to produce archegonia (Huang et al., 2004; Quintanilla et al., 2007; DeSoto et al., 2008). In *O. cinnamomeum*, culture densities of <4 spores cm^−2^ produced exclusively female gametophytes, with males only becoming common at much higher densities (Huang et al., 2004). In the Hornych et al. (2021) study of *C. multiflora*, densities were even lower, at approximately 0.32 spores sown per square centimeter. This low density may have strongly influenced the sex expression of *C. multiflora* in culture. We observed similarly low densities in our field study, with total densities ranging from approximately 1.8 gametophytes cm^−2^ to 8.1 gametophytes cm^−2^, suggesting that the formation of unisexual females in *C. multiflora* gametophyte colonies may be closely related to competition and resource availability.

A potential challenge to our determination that *C. multiflora* possesses a putative antheridiogen system is that as more spores germinate, realized density of the colony increases over time; therefore, there would naturally be some progression from females among initially germinating spores to males as more spores germinated and competition increased. However, if this were the case, then we would not predict male sex expression to be related to the presence of females as we demonstrated in our study. In the study of Hornych et al. (2021), the density was the same in control and treatment colonies, yet antheridia only initiated first in the group treated with a mature gametophyte. Thus, although direct competition may partially explain the formation of males under dense conditions, several lines of evidence including the confirmation of both of our predictions in two of three colonies and the initiation of antheridia exclusively in the presence of females reported by Hornych et al. (2021) provide evidence for an antheridiogen system in *Cyathea multiflora*.

### Other considerations

This study explored two predictions on the function of antheridiogen in natural gametophytic colonies. One limitation is that we were only able to sample three colonies of gametophytes. Such sampling was partially due to sampling difficulty. Separating, mounting, and identifying this number of gametophytes was time consuming, so a larger sample size was not logistically feasible. We also spent considerable time seeking colonies that shared common ecological variables. Defining a singular colony is not a simple task, and we did not want to split up colonies into smaller groups when interaction between them would have been confounding. Additionally, the overall diversity and richness of our study site led us to conservatively define fewer colonies with more gametophytes than in other field studies (e.g., Hamilton and Lloyd, 1991, average of 27.7 gametophytes per colony vs. 107.3 per colony in the present study). While this sampling reduces our ability to test our predictions across many gametophytic colonies, it does allow us to detect spatial patterns more robustly within them. Differences in microclimate, resources, and soil that affect sex expression also cannot be completely ruled out as factors influencing sex expression. While local environmental conditions at the site may influence sex expression across all three colonies, the consistency in sex distribution at the single colony scale indicates there were not stark differences in microclimates between them. Finally, additional compounds may be involved in a wide‐array of intra‐ and interspecific interactions between fern gametophytes because some studies have documented allelopathic‐type effects of fern gametophytes on each other even when antheridiogen is absent (Petersen and Fairbrothers, 1980; Testo et al., 2014; Hornych et al., 2022). These interactions often result in severe reductions in growth and cessation of development altogether, preventing even the formation of antheridia, so are not likely the cause of males in our study.

### Future directions

Given the results of the present study, follow‐up studies on sex expression in *C. multiflora* are clearly needed. Future work should track the size and sex expression of gametophytes over time in different sowing densities and culture conditions across *C. multiflora* compared to *Cyathea* species without antheridiogen. Additional work in the Cyatheales is also encouraged because of the apparent diversity in sex‐expression patterns and gametophyte responses to the environment among families in this group (Stokey, 1930; Nayar and Kaur, 1971; Hornych et al., 2021). Given that the Cyatheales are derived relative to the earlier‐branching Schizaeles where regular antheridiogen systems first appear (Greer et al., 2009), but are sister to the core leptosporangiate clades within which antheridiogen systems are highly refined, the antheridiogen system of this group may be at an intermediate stage of evolution.

## CONCLUSIONS

We provide the first evidence of antheridiogen function in natural colonies of *Cyathea multiflora* gametophytes. Our study adds to the limited work done on antheridiogen activity in situ and provides field confirmation of the surprising recent discovery of antheridiogen systems in *Cyathea*. We confirmed two predictions about antheridiogen function in natural colonies: (1) The distribution of sex expression is male‐biased and hermaphrodites are uncommon. (2) Male sex expression is a function of female proximity. Importantly, these predictions are useful for identifying antheridiogen in natural colonies, and we strongly encourage continued study of fern gametophytes in the field.

## AUTHOR CONTRIBUTIONS

J.E.W. and A.D.H. designed the study and collected the data. A.D.H. and J.B.M. analyzed the data. A.D.H. wrote the manuscript with substantial input from J.B.M. and J.E.W.

## Data Availability

The authors commit to open access for all data and analyses. Raw data files and R scripts used are available at the Dryad Digital Repository: https://datadryad.org/stash/share/qkw78eRI15GGZW397E26O21ltJ2rLyZOaUl85UCrbac (Harrington et al., 2022).
